# Microbiological testing of devices used in maintaining peripheral venous catheters**^1^**

**DOI:** 10.1590/1518-8345.1528.2887

**Published:** 2017-05-15

**Authors:** Fernanda de Paula Rossini, Denise de Andrade, Lissandra Chaves de Sousa Santos, Adriano Menis Ferreira, Caroline Tieppo, Evandro Watanabe

**Affiliations:** 2 PhD, RN, Hospital das Clínicas, Faculdade de Medicina de Ribeirão Preto, Universidade de São Paulo, Ribeirão Preto, SP, Brazil.; 3 PhD, Associate Professor, Escola de Enfermagem de Ribeirão Preto, Universidade de São Paulo, WHO Collaborating Centre for Nursing Research Development, Ribeirão Preto, SP, Brazil.; 4 Doctoral student, Escola de Enfermagem de Ribeirão Preto, Universidade de São Paulo, WHO Collaborating Centre for Nursing Research Development, Ribeirão Preto, SP, Brazil.; 5 PhD, Associate Professor, Universidade Federal do Mato Grosso do Sul, Três Lagoas, MS, Brazil.; 6 Pharmacy-Biochemistry, Hospital Regional do Mato Grosso do Sul, Campo Grande, MS, Brasil.; 7 PhD, Professor, Faculdade de Odontologia de Ribeirão Preto, Universidade de São Paulo, Ribeirão Preto, São Paulo, Brasil.

**Keywords:** Cross Infection, Vascular Access Devices, Catheters, Contamination, Bacteria, Drug Resistance, Microbial

## Abstract

**Objective:**

to evaluate the use of peripheral venous catheters based on microbiological analysis of devices (dressing and three-way stopcocks) and thus contribute to the prevention and infection control.

**Methods:**

this was a prospective study of microbiological analysis of 30 three-way stopcocks (external surfaces and lumens) and 30 dressing used in maintaining the peripheral venous catheters of hospitalized adult patients.

**Results:**

all external surfaces, 40% of lumens, and 86.7% of dressing presented bacterial growth. The main species isolated in the lumen were 50% coagulase-negative *Staphylococcus*, 14.3% *Staphylococcus aureus*, and 14.3% *Pseudomonas aeruginosa.* Fifty nine percent of multidrug-resistant bacteria were isolated of the three-way stopcocks, 42% of the lumens, and 44% of the dressing with a predominance of coagulase-negative *Staphylococcus* resistant to methicillin. Besides, 18% gram-negative bacteria with resistance to carbapenems were identified from multidrug-resistant bacteria on the external surfaces of the three-way stopcocks.

**Conclusion:**

it is important to emphasize the isolation of coagulase-negative *Staphylococcus* and gram-negative bacteria resistant to methicillin and carbapenems in samples of devices, respectively, which reinforces the importance of nursing care in the maintenance of the biologically safe environment as well as prevention and infection control practices.

## Introduction

Health care in the hospital environment is constantly challenged by infections, which result in increases in morbidity and mortality, length of stay, and costs, especially considering the consumption of antibiotics and laboratory tests. Given the proportions, these infections represent one of the largest public health problems, which is aggravated by the presence of resistant strains, since they challenge scientific and technological advances and therefore catch the attention of professionals, researchers, and organizations looking for effective prevention and control measures^1^ ^-^ ^3^.

Risk factors for infection is an indicator that deserves careful analysis and elucidation in the management of aseptic techniques. This analysis represents a challenge, given variability of risks and diversity of behaviors and recommendations. In this sense, the need emerges to objectively identify the possible risks of contamination and colonization^4^ ^-^ ^5^.

The use of intravascular catheters constitutes a vital strategy for clinical practice and effectiveness of treatments^6^ ^-^ ^8^. It is worth mentioning that peripheral venipuncture is not free of complications, since it is an invasive procedure of high frequency done most of the time in a hospital setting, which is a place that presents risks of contamination and colonization, and which requires appropriate conduct in care of catheters. 

The maintenance of peripheral venous catheters is a complex topic and requires following a number of technical aseptic conformities and operational principles with a view toward safety and the prevention and control of infection. 

The incidence of phlebitis and infections associated with peripheral venous catheters is relatively low, but the possibility of underestimation of data should be pointed out, especially considering the high frequency with which this procedure is done in daily routine health care. Catheter-related bacterial infections prolong hospitalization and increase the cost of treatment, besides presenting attributable mortality rates in the range between 10% to 25%^6^ ^,^ ^9^.

According to these questions: Is it possible to isolate bacteria from samples of devices (dressing and three-way stopcocks - 3WSCs) used in peripheral venous access? What is the prevalence and sensitivity profile of the isolated bacteria? Moreover, are these bacteria resistant to carbapenems?

Therefore, this research attempts to evaluate the microbiological conditions of devices (dressing and 3WSCs) used in peripheral venous access and thus, contribute to the prevention and control of infection.

## Methods

This is a microbiological clinical study done on the 3WSCs and dressing used in the maintenance of peripheral venous catheters (PVC) type abbocath used in hospitalized patients.

Samples from the devices were collected for adult patients admitted to the medical and neurological clinical specialty of a public university hospital inpatient for highly complex clinical emergency care. For 60 devices (30 dressing and 30 3WSCs), 90 microbiological processes were carried out, 30 from the external surfaces, 30 from the lumens of the 3WSCs, and 30 from the dressing. The devices analyzed were collected after discontinuing use of the intravenous devices (due to doctor's orders, obstruction, infiltration, the presence of classic phlebitis signs of pain, edema, hyperthermia, and local hyperemia), or in cases where there was a need to change the dressing of the access considering the conditions of integrity and humidity. The removal of these devices from the patients was done exclusively by nurses working in the unit. We believe that removal according to hospital routine preserved the microbiological conditions of the real situation of hospital care. Any type of contamination was avoided during the removal and transfer of the samples to their sterile packaging. The area of the dressing in contact with the catheter insertion site was marked externally to show where the microbiological collection should be made.

Furthermore, information was collected as to the date of the peripheral venipuncture that was on the dressing and description of the general conditions in terms of dirtiness or presence of blood (evaluation of the macroscopic condition of the dressing). The research was conducted with the approval of the Research Ethic Committee (37194214.1.0000.5393).

### Inclusion criteria

The evaluation included 3WSCs used in maintaining the PVC and sterile dressing made of soft fabric backed with rayon and polyester with hypoallergenic acrylate adhesive, water-resistant, non-owcclusive, and made of hypoallergenic transparent film with vapor permeability.

### Microbiological processing

The collection of biological material from the dressing was done by friction on the inner surface with a swab moistened with saline solution, which was in contact with the catheter insertion site (previously defined area) for 30 seconds and in three directions. Then the swab was transferred to a sterile test tube (25 mm x 125 mm) containing 20 mL of *Tryptic Soy Broth* (TSB). For the culture from the 3WSCS's lumen, a syringe and sterile gloves were used to carry out a flush of 10 mL of TSB through each of the two routes of the 3WSCS into a sterile test tube (25 x 125 mm) with glass beads. For the culture of the external surface the 3WSCS was flushed with the routes closed into a sterile vial with 200 mL of TSB.

After these procedures, homogenization was carried out on the samples using an AP-56 tube shaker (Phoenix, Brazil) during 1 minute and then the samples were incubated (Quimis, Brazil) at 37°C for 24 hours up to 14 days (sterility test). The initial microbiological analyses were performed in a Class II Biological Safety Cabin - Model Bio Seg 12 (VECO Group, Brazil) in the microbiology laboratory. After incubation, the samples were seeded on Petri plates (15 x 90 mm) with selective culture media (Mannitol, MacConkey, and Cetrimide) and processed in a VITEK^(r)^ 2 Compact automated system (Biomérieux, France) for bacterial identification and sensitivity profile.

## Results

Of the total of 90 microbiological analyses, the samples from the lumen of the 3WSCs had growth positive levels of 40% in the TSB culture medium. The samples from the dressing showed 86.7% of contamination and the external surface of the 3WSCs 100% (Table 1).

**Table 1:** Evaluation of bacterial growth on dressing, lumens and external surfaces of the three-way stopcocks used in peripheral venous catheters. Ribeirão Preto, SP, Brazil, 2015

Devices	Bacterial Growth	Culture Medium (***Tryptic Soy Broth*** )	
		n	%
Dressing	Present	26	86.7
	Absent	4	13.3
Three-way stopcocks lumen	Present	12	40.0
	Absent	18	60.0
Three-way stopcocks external surfaces	Present	30	100
	Absent	0	0

The length of stay of the PVC ranged from 2 to 8 days: 36.7% of the samples indicated that the venous catheterization had been done three days ago (72 hours) and 26.7% four days ago (96 hours). The mean and median of the length of stay of the PVC was 3.75 days and three days respectively with a standard deviation of 1.48.

Additionally, when the presence or absence of macroscopic dirtiness on the devices was analyzed, such as the presence of blood with bacterial growth in a TSB culture medium, it was observed that 46.6% of these samples were not considered dirty but had a positive culture for bacterial growth, 28% were considered dirty and showed positive results, 16.7% were considered dirty but were not positive, and 7.8% were not considered dirty and also were not positive.

**Figure 1:**
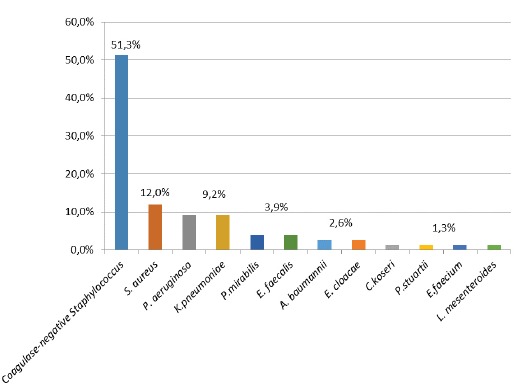
Evaluation of bacteria distribution in the samples of dressing, lumens and external surfaces of the three-way stopcocks used in peripheral venous catheters. Ribeirão Preto, SP, Brazil, 2015

As for the microbiological evaluation of the devices (dressing, lumen, and the external surface of the 3WSCs), 76 bacteria were isolated from the total of 68 positive samples for bacterial growth with the main ones being as follows: coagulase-negative *Staphylococcus* at 51.3%, *Staphylococcus aureus* at 12%, *Pseudomonas aeruginosa* at 9.2%, *and Klebsiella pneumoniae* at 9.2% (Figure 1).

Regarding the evaluation of the profile of sensitivity to antibiotics of the bacteria isolated in the positive samples of the dressing, lumen, and external 3WSCS surface, 44%, 35.7%, and 73.3% showed growth of multi-resistant microorganisms, respectively. It should be pointed out that in some samples more than one bacterium was isolated.

Of the total of resistant microorganisms isolated in the samples of the culture from the dressing, the two that stand out the most are coagulase-negative *Staphylococcus* resistant to methicillin at 91% and *Klebsiella pneumoniae* resistant to carbapenems at 9% (Table 2).

**Table 2:** Evaluation of numerical and percentage distribution of bacteria in dressing used in peripheral venous catheters according to antibiotics sensitivity profile. Ribeirão Preto, SP, Brazil, 2015

Bacteria	Dressing				
	Resistant (n=11)				Sensitive (n=14)
	n	%		n	%
Coagulase-negative *Staphylococcus*	10*	91.0		3	21.4
*Pseudomonas aeruginosa*	0	0		3	21.4
*Klebsiella pneumoniae*	1**^†^**	9.0		2	14.3
*Staphylococcus aureus*	0	0		2	14.3
*Enterobacter cloacae*	0	0		2	14.3
*Proteus mirabilis*	0	0		1	7.10
*Leuconostoc mesenteroides*	0	0		1	7.10

* Methicillin-resistant coagulase-negative *Staphylococcus.*

† Carbapenem-resistant *Klebsiella pneumoniae*.

In the lumen of the 3WSCs (Table 3), the one that stands out the most is coagulase-negative *Staphylococcus* resistant to methicillin at 100%.

**Table 3:** Evaluation of numerical and percentage distribution of bacteria in lumens of the three-way stopcocks used in peripheral venous catheters according to antibiotics sensitivity profile. Ribeirão Preto, SP, Brazil, 2015

Bacteria	3WSCs* lumen				
	Resistant (n=5)				Sensitive (n=9)
	n	%		n	%
Coagulase-negative *Staphylococcus*	5^*^	100		2	22.2
*Staphylococcus aureus*	0	0		2	22.2
*Pseudomonas aeruginosa*	0	0		2	22.2
*Klebsiella pneumoniae*	0	0		1	11.0
*Proteus mirabilis*	0	0		1	11.0
*Enterococcus faecalis*	0	0		1	11.0

* Methicillin-resistant coagulase-negative *Staphylococcus.*

Of the total of resistant bacteria isolated in the samples from the external surface of the 3WSCs (Table 4), the three that stand out the most are coagulase-negative *Staphylococcus* with a 72.7% prevalence, followed by 9% for *Staphylococcus aureus* resistant to methicillin, and 9% for *Klebsiella pneumoniae* resistant to carbapenems.

**Table 4:** Evaluation of numerical and percentage distribution of bacteria in external surfaces of the three-way stopcocks used in peripheral venous catheters according to antibiotics sensitivity profile. Ribeirão Preto, SP, Brazil, 2015

Bacteria	3WSCs* external surface				
	Resistant (n=22)			Sensitive (n=15)	
	n	%		n	%
Coagulase-negative *Staphylococcus*	16^*^	72.7		3	20.0
*Staphylococcus aureus*	2^*^	9.0		3	20.0
*Klebsiella pneumoniae*	2**^†^**	9.0		1	6.60
*Pseudomonas aeruginosa*	1**^†^**	4.5		1	6.60
*Acinetobacter baumannii*	1**^†^**	4.5		1	6.60
*Enterococcus faecalis*	0	0		2	13.3
*Citrobacter koseri*	0	0		1	6.60
*Proteus mirabilis*	0	0		1	6.60
*Providencia stuartii*	0	0		1	6.60
*Enterococcus faecium*	0	0		1	6.60

* Methicillin-resistant (coagulase-negative *Staphylococcus* and *Staphylococcus aureus*)*.*

† Carbapenem-resistant (*Klebsiella pneumoniae, Pseudomonas aeruginosa* and *Acinetobacter baumannii).*

## Discussion

The use of intravascular catheters is one of the important advances in medicine, but we should not be oblivious to the risks inherent in their use, especially infectious events. Some factors that increase the risk of infection are use of the devices, insertion site, length of stay, skin preparation, and type and way of fastening the dressing used^8^ ^-^ ^10^. There is a further concern with the 3WSCs, because beyond being useful in peripheral venous infusions, they also belong to the arsenal of venous extensions. 

The literature reveals a low risk of infection of the bloodstream associated with the use of peripheral venous catheters; however, we must consider its high use in health care, a fact that has been changing this scenario^8^. On the other hand, one study^11^ demonstrated that the number of bloodstream infections caused by peripheral venous catheters and central venous catheters were similar, and that the peripheral catheters inserted in the emergency department caused the largest number of episodes.

The study evaluated the microbiota, the profile of sensitivity of the bacteria isolated from some devices (dressing, lumens, and external surfaces of the 3WSCS) used in the maintenance of peripheral venous access, the duration of use of the device, and the presence of macroscopic dirtiness. 

In the study, microbial growth in the cultures from the external surfaces of the 3WSCs was 100%. Some investigators have speculated that the surface contamination of the 3WSCS occurs due to environmental exposure, manipulation by nursing staff, contact with the patient's microbiota and contact with the bed linen^8^ ^,^ ^12^. Among the resistant bacterial species isolated from the 3WSCS surfaces, the main ones were gram-negative bacteria with a resistance to carbapenems such as *Klebsiella pneumoniae* (9%), *Pseudomonas aeruginosa* (4.5%), and *Acinetobacter baumannii* (4.5%). Thus, it is important to emphasize that good aseptic practices should be carefully adopted and enforced by the professionals who handle the devices used in the maintenance of the venous catheters in order to avoid contamination of the internal lumen and resultant bloodstream infections^8^ ^,^ ^10^.

At the center of discussions of infection control in health services is the behavior of health professionals, and it is mentioned as an important tool in the implementation of safe practices^1^ ^,^ ^13^. But even though measures to prevent and control bloodstream infection are carefully established by guidelines, the reality of health care points to unsatisfactory levels of compliance by health professionals, especially for the practices of washing hands (10.7%) and disinfection of hubs and connectors (40.0%) prior to drug administration^10^ ^,^ ^14^. It should be noted that connections such as 3WSCs/hubs and infusion equipment are a common gateway for microorganisms^15^. Inadequate disinfection of connectors can result in bacterial contamination of the inner lumen of the catheter, resulting in the formation of biofilm and subsequent bloodstream infection^8^. On the other hand, we cannot ignore the possibility that contamination of the catheter's inner lumen may originate in the migration of bacteria inside the catheter coming from the patient's skin and migrating to the catheter's tip.

We consider the contamination rate in the lumen of the 3WSCs (40%) to be high and worrisome, especially by resistant bacteria such as coagulase-negative *Staphylococcus* resistant to methicillin. In general, for hospitalized patients undergoing intravenous therapy, the devices are frequently manipulated to administer drugs at regular intervals; or as is the case with antibiotics, every 6 hours; or analgesics and antipyretics in the case of pain or fever, among other drugs. This fact reinforces the need for professionals to adopt safe practices when administering drugs, including meticulous hand hygiene.

Thus, we highlight the importance of disinfecting the hubs and hand hygiene (HH) before and after handling the devices, along with other procedures that impede contamination of the lumen on the 3WSCs. So failure to observe aseptic principles contributes to contaminating the devices. 

HH is the simplest and least expensive individual measure to prevent the spread of healthcare-related infections^6^. However, it was not our objective in this study, to determine the frequency of this practice when handling the device.

Furthermore, the lids should be used when closing the 3WSCs, which should be strictly maintained in order to preserve their sterile condition. However, in the real situation of providing care, actions can be taken that contaminate the inside of the lids, such as leaving them on trays or other surfaces with the inner side facing down and then reusing them, when in reality the 3WSCs lids should be discarded with every handling of the device for infusion^10^.

Furthermore, we observed that 86.7% of the culture from the dressing in TSB were positive, which is contrary to our hypothesis. It was speculated that it would be 100%, since dressing are in direct contact with the patient's skin and their endogenous microbiota, and mainly because the sample is collected at the catheter's insertion^11^. 

The risk became alarming when profile of sensitivity to antibiotics of the species were analyzed; coagulase-negative *Staphylococcus* resistant to methicillin was at 91% and *Klebsiella pneumoniae* resistant to carbapenems was at 9%. It should be noted that perhaps four (13.3%) of the negative cultures were associated with the technique of swab collection at just one point (ostium of insertion), duration of use of the dressing, humidity control, and best aseptic practices^16^.

The purpose of the dressing is to protect the puncture site and minimize the possibility of infection through the interface between the surface of the catheter and the skin. The dressing should be replaced immediately if contamination is suspected and always when wet, loose, dirty, or with compromised integrity. It is important to protect the insertion site with plastic when showering with a dressing that is not waterproof^8^ ^,^ ^10^. It should be pointed out that in this study the dressing was waterproof.

In the etiology of hospital infections, the presence of resistant strains has had an impact on morbidity, mortality, and costs, reaching proportions that are very worrisome^3^ ^,^ ^17^. The participation of *S. aureus* resistant to methicillin and of gram-negative bacilli resistant to carbapenems is increasingly frequent in episodes of bacteremia in critically ill patients^18^. Thus, another challenging result involved evaluating multidrug-resistant strains and strains with resistance to carbapenems in the maintenance devices of peripheral venous catheters. For decades, the world has witnessed a proliferation of microbes with antibiotic resistance, which implies the acquisition of genes that determine resistance to the point of becoming refractory to virtually all antibiotics, leaving researchers and health professionals in a bleak environment with no therapeutic options. Undoubtedly, one of the most important factors involved is the wide use of antibiotics in outside of hospitals.

Another important point is that in health institutions, the spread of resistant strains is also facilitated by noncompliance with basic recommendations such as washing hands, use of protective barriers, and decontamination of equipment, among other practices^19^.

Although there is evidence in literature of low risk of local infection of the bloodstream associated with peripheral venous catheters, this situation is changing^9^. For this reason, one should not miss the importance of this issue, especially considering its gravity, the etiology of the microbial species, and the main predisposing factors^20^ ^-^ ^21^. The success of the fight against infection depends not only on accurate diagnosis, but also, and in the same proportion, on improvement of conditions in terms of infrastructure and human resources. As mentioned before, the performance and attitudes of health professionals is relevant. It is necessary to begin to build an educational system that promotes knowledge, skills, and attitudes that are converted into legitimized critical and reflective professional practice. Some of these issues are more acute in countries facing crises in basic conditions of infrastructure, which includes the training of human resources.

The results of the present study encourage a number of reflections, and one is the need to perform essential prospective surveillance to prevent and control hospital infections, thus favoring decision-making based on situations of real care.

This research presents limitations on the reduced size of the samples, which allowed only the analysis of the data by descriptive statistics. On the other hand, the results provide a basis for raising awareness about the importance of following safe practices when providing care for patients with intravenous devices. One result could be the instigation of the development of future clinical research concerned mainly with the relationship among: duration of use of peripheral venous catheters, sensitivity profiles, genetic similarity among the various sample collection locations (3WSCS external surfaces and lumens, dressing, and insertion sites), and the biofilm formation on the devices.

## Conclusion

In general, the results are alarming because the contamination by antimicrobial resistant bacteria was identified, and coagulase-negative *Staphylococcus* resistant to methicillin was predominant in samples from dressing, lumen and the external surface of 3WSCS. Moreover, it is important to emphasize the isolation of gram-negative bacteria resistant to carbapenems on dressing and external surface of 3WSCS, due to the pathogenicity of these microorganisms, which reinforces the importance of nursing care in the maintenance of the biologically safe environment as well as prevention and infection control practices.
